# Neuropeptide B promotes proliferation and differentiation of rat brown primary preadipocytes

**DOI:** 10.1002/2211-5463.13128

**Published:** 2021-03-12

**Authors:** Tatiana Wojciechowicz, Maria Billert, Priyavathi Dhandapani, Dawid Szczepankiewicz, Oskar Wasielewski, Mathias Z. Strowski, Krzysztof W. Nowak, Marek Skrzypski

**Affiliations:** ^1^ Department of Animal Physiology, Biochemistry and Biostructure Poznan University of Life Sciences Poland; ^2^ Department of Hepatology and Gastroenterology Charité‐University Medicine Berlin Germany; ^3^ Department of Zoology Poznan University of Life Sciences Poland; ^4^ Department of Internal Medicine‐Gastroenterology & Oncology Park‐Klinik Weissensee Berlin Germany

**Keywords:** adipogenesis, brown adipose tissue, differentiation, neuropeptide B, preadipocytes, proliferation

## Abstract

Neuropeptide B (NPB) is reported to regulate energy homeostasis and metabolism via the NPBWR1 and NPBWR2 receptors in various tissues. However, the molecular mechanisms triggered from their interaction are not well investigated in brown adipose tissue. In this study, we specifically analyzed the role of NPB in controlling brown adipogenesis in rat brown preadipocytes. We first detected the expression of NPBWR1 and NPB on mRNA and protein level in brown preadipocytes and observed that NPB increased viability and proliferation of preadipocytes. Moreover, NPB stimulated expression of adipogenic genes (*Prdm16*, *Ucp1*) and suppressed the expression of antiadipogenic preadipocyte factor 1 (*Pref1*) during the differentiation process. Altogether, this led to an increase in intracellular lipid accumulation during preadipocyte differentiation, coupled with an increase in adrenaline‐induced oxygen consumption mediated by NPB. Furthermore, *Ucp1* expression stimulated by NPB was attenuated by blockade of p38 kinase. In summary, we conclude that NPB promotes proliferation and differentiation of rat brown preadipocytes via p38‐dependent mechanism and plays an important role in controlling brown adipose tissue formation.

## Graphical abstract

Neuropeptide B (NPB) and NPBWR1 are expressed in brown preadipocytes. NPB increased viability and proliferation of preadipocytes. NPB stimulated expression of adipogenic genes (*Prdm16*, *Ucp1*) and lipid accumulation. *Ucp1* expression stimulated by NPB was attenuated by blockade of p38 kinase. We conclude that NPB modulates brown adipogenesis via p38‐dependent mechanism.

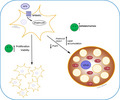

**Abbreviations**
BrdU: 5‐bromo‐2’‐deoxyuridine
DMEM/F12: Dulbecco’s modified Eagle’s medium/F12
ERK1/2: extracellular signal regulated kinases 1/2
Hprt1: hypoxanthine phosphoribosyl transferase
HRP: horseradish peroxidase
NPB: neuropeptide B
ORO: oil red O
Pgc1α: peroxisome proliferator‐activated receptor gamma coactivator 1‐alpha
Pparγ: peroxisome proliferator‐activated receptor gamma
Prdm16: PR domain containing 16
Ucp1: uncoupling protein 1

Neuropeptide B (NPB) is a hormone peptide composed of 29 amino acids uniquely modified with bromine at the N‐terminal tryptophan residue [1]. Biological effects of NPB are mediated through activation of two G protein‐coupled receptors, termed as NPBWR1 (GPR7) and NPBWR2 (GPR8) [1, 2]. In humans, both NPBWR1 and NPBWR2 are expressed, while in rodents NPBWR2 expression has not been so far described [2].

Neuropeptide B is expressed in the brain [1, 3, 4] as well as in peripheral tissues such as heart, gastrointestinal system, pancreatic islets, and fat [4, 5]. NPB was implicated in controlling appetite [2], stress hormone secretion [6], stimulation of aldosterone release [7], and inflammatory pain modulation [8]. Recently, we found that NPB may contribute to glucose homeostasis by suppressing insulin expression and secretion in pancreatic beta cells [9]. Moreover, there is growing evidence indicating that NPB and its receptors are involved in controlling energy homeostasis and body weight regulation. For example, it was found that genetic depletion of NPBWR1 in male mice leads to adult‐onset obesity and impaired energy expenditure [10]. Additionally, the same animals had elevated levels of glucose, insulin, and leptin in the circulation [10]. Consistently, male NPB ‐deficient mice (NPB −/−) developed adult‐onset obesity [8]. Furthermore, it was found that NPB may be involved in controlling white adipocytes metabolism and endocrine activities. We reported recently that by acting on white adipocytes, NPB suppresses expression and secretion of leptin, and promotes lipolysis [11]. These results collectively suggest that NPB may contribute to whole‐body metabolism and body weight regulation by modulating white adipocytes functions. In contrast to white fat tissue, brown adipocytes protect from obesity by promoting energy expenditure via UCP1‐dependent manner [12]. It is important to note that the loss of brown adipocytes is accompanied by obesity and decreased energy expenditure, which resemble metabolic abnormalities descripted in NPB‐ or NPBWR1‐deficient mice [8, 10]. Since the role of NPB in controlling brown adipose tissue functions is still unknown, we therefore evaluated the effects of NPB on proliferation and differentiation of rat primary brown preadipocytes into mature brown adipocytes.

## Materials and methods

### Reagents

Rat (Des‐Br)‐ NPB‐29 was purchased from Phoenix Pharmaceuticals (Burlingame, CA, USA). Cell culture media and FBS were from Biowest (Nuaillé, France). 3‐(4,5‐Dimethyl‐2‐thiazolyl)‐2,5‐diphenyl‐2H‐tetrazolium bromide (MTT) was from Calbiochem (Merck, Darmstadt, Germany). The 5‐bromo‐2′‐deoxyuridine (BrdU) Cell Proliferation ELISA kit was from Roche Diagnostics (Basel, Switzerland). Phospho‐ERK1/2 (cat. no. 9101S), extracellular signal regulated kinases 1/2 (ERK1/2; cat. no. 9102S), phospho‐p38 MAPK (cat. no. #9211), p38 MAPK (cat. no. #9212), and antibodies and Anti‐rabbit IgG horseradish peroxidase (HRP)‐linked antibody (cat. no. #7074) were from Cell Signaling Technology (Danvers, MA, USA). Rabbit Anti‐UCP1 antibody (ab209483) was from Abcam (Cambridge, UK). Anti‐NPBWR1 antibody (cat. no. bs‐8618R) was from Bioss Antibodies (Wobur, MA, USA). Anti‐NPB antibody (cat no. LS‐C150319) was from Lifespan Biosciences (Seattle, WA, USA). Anti β‐actin (A1978) was from Sigma‐Aldrich (St. Louis, MO, USA). Other reagents were purchased from Sigma‐Aldrich, unless otherwise stated.

### Cell isolation and culture

The isolation of primary brown preadipocytes was carried out as described previously [13, 14]. Rat brown preadipocytes were isolated as stromal‐vascular fraction of interscapular fat pad cut from male Wistar rats (100–120 g). All procedures concerning welfare of animals agreed with the ethical standards set by the National Ethics Commission for Investigations on Animals in Poland. Briefly, interscapular fat was isolated immediately after rats decapitation, cleaned from adjacent tissues (e.g., muscles), and placed in Krebs‐Ringer buffer (118 mm NaCl, 4.8 mm KCl, 1.3 mm CaCl_2_, 1.2 mm KH_2_PO_4_, 1.2 mm MgSO_4_, 24.8 mm NaHCO_3_, 5 mm glucose, 3% BSA) with antibiotics (100 U·mL^−1^ penicillin and 0.1 mg·mL^−1^ streptomycin), under sterile conditions. After three‐time washing, fat tissue was cut into small pieces with scissors and placed in prewarmed collagenase type II (3 mg·mL^−1^; Sigma‐Aldrich) solution. The collagenase digestion was performed for 60 min in a water bath (37 °C) with shaking every 10 min. Next, digestion mixture was diluted with Krebs‐Ringer buffer and the suspension was centrifuged 10 min (450 ***g***, RT). The pellet containing preadipocytes was cleaned from erythrocytes using Red Blood Cell Lysing buffer (Sigma‐Aldrich) incubation (5 min with gentle pipetting). After lysis of erythrocytes, Krebs‐Ringer buffer was added and inoculum was filtrated through a 100‐µm nylon mesh. Then, it was incubated for 10 min to allow vascular endothelium cells aggregation and finally filtrated through a 45‐µm nylon mesh. After the final centrifugation (450 ***g***, 10 min, RT), preadipocytes‐enriched pellet was resuspended in Dulbecco's modified Eagle's medium/F12 (DMEM/F12) supplemented with 10% FBS and antibiotics (penicillin‐streptomycin). Cells were counted, and viability was estimated using 0.4% trypan blue dye (viability over 95% was acceptable). Cells were then seeded into culture plates. Cell cultures were carried out in a humidified incubator with atmosphere consisting of 95% air and 5% CO_2_ (37 °C) for 24 h. Then, cells were washed with warm PBS, fresh culture medium was added, and the below described experiments were performed.

### Cell viability and proliferation

Cell viability and proliferation were analyzed as described previously [15]. In brief, preadipocytes (4 × 10^3^ cells/well of 96‐well plates) were precultured for 24 h, washed with warm PBS solution and cultured in DMEM/F12 medium containing antibiotics in the presence or absence of NPB (1, 10, and 100 nm) for 24 h. Viability of the brown preadipocytes was measured by adding 10 µL/well of MTT solution (5 mg·mL^−1^ in PBS) for 30 min. Next, medium was removed and formed formazan crystals were dissolved in 100 µL of DMSO. The absorbance was determined at 570 nm wavelength against isopropanol (blank) using microplate reader Synergy 2 (BioTek, Winooski, VT, USA). Cell proliferation was studied using a Cell Proliferation ELISA BrdU colorimetric kit according to manufacturer’s instruction, as described previously [16].

### Differentiation of brown preadipocytes

Preadipocytes (100% confluency) were differentiated into mature adipocytes by incubating them in the presence of a differentiation medium [DMEM/F12 supplemented with 0.5 mm isobutyl methylxanthine, 1 µg·mL^−1^ insulin, and 2 nm triiodothyronine (T3)] in the presence or absence of NPB. Differentiating medium was changed every 2 days.

### Oil red O staining

In brief, cells were washed with warm PBS and fixed with 10% formaldehyde in PBS for 10 min. Next, freshly prepared 10% formaldehyde was added and cells were incubated for 1 h at RT. Working oil red O (ORO) solution was freshly prepared by mixing six parts of ORO stock solution (0.7 g per 200 mL isopropanol) with four parts of distilled water. After 20 min of incubation at RT, ORO working solution was filtrated through 0.22‐µm syringe filter and added to previously washed (60% isopropanol) and dried cells and allowed to react for 10 min at RT. Next, cells were washed four times with distilled water and microscopic documentation was done using LSM 510‐inverted microscope and axio vision rel. version 4.6 software (Carl Zeiss, Obekochen, Germany). After microphotographing, cells were dried and ORO was eluted by adding 100% isopropanol. For quantification, eluates were transferred into 96‐well plates and the absorbance was measured at a wavelength of 520 nm using a Synergy 2 Multi‐Mode Microplate Reader (BioTek Instruments, Inc., Winooski, VT, USA).

### Reverse transcription and Real‐time qPCR

Total RNA was isolated using Extrazol reagent (DNA‐Gdańsk, Gdańsk, Poland) according to manufacturer's instruction. Extracted RNA was analyzed was measured using NanoDrop 1000 spectrophotometer (Thermo Fisher Scientific, Waltham, MA, USA). One microgram of total RNA was reverse transcribed to cDNA using FIREScript RT cDNA Synthesis Mix (Solis BioDyne, Tartu, Estonia) with Oligo (dT) and Random primers. The real‐time quantitative PCRs contained HOT FIREPol EvaGreen qPCR Mix Plus (Solis BioDyne) and were performed using QuantStudio 12K Flex (Thermo Fisher Scientific). The primers were purchased from Sigma‐Aldrich; their sequences are listed in Table 1. The results were calculated using double delta CT method. Expressions of target genes were normalized to the expression level of hypoxanthine phosphoribosyl transferase *Hprt* mRNA.

**Table 1:** Sequences of PCR primers.

Gene	Left primer (5’ > 3’)	Right primer (5’ > 3’)	NCBI Reference Sequence
*Npb*	tttccacaggttcccatcc	agttgcgcagaggtacgg	NM_153293.1
*Npbwr1*	gaggcacacagccagaatc	ctggaccgtgccaagaag	XM_032890525.1
*Hprt1*	cagtcaacgggggacataaaag	attttggggctgtactgcttga	XM_032889905.1
*Prdm16*	gtgcctactccttgggtgtc	gaagagctcatcacactcatcg	XM_017593884.1
*Pparγ*	caggaaagacaacagacaaatca	gggggtgatatgtttgaacttg	NM_013124.3
*Ucp1*	gcctgcctagcagacatcat	tggccttcaccttggatct	NM_012682.2
*Pref1*	atatgccaccaccccctac	gggcaaggagctttaactctg	AB046763.1

### Western blot

Cells were seeded on 6‐well plates, precultured 24 h, washed and cultured for 3–4 days to 100% confluence. Next, differentiating medium with or without NPB (10, 100 nm) was added and at day 1 (for UCP1 analysis) or at time points (0, 5, 10, 15, and/or 30, 60 min; for phosphorylation of ERK1/2 or p38). Cells were washed with iced‐cold PBS, and RIPA lysis buffer (Sigma‐Aldrich) containing phosphatase and protease inhibitor cocktails (Roche Diagnostics) was added, and cells were incubated for 5 min on ice. Thereafter, lysates were centrifuged (14 000 ***g***, 10 min, 4 °C). Supernatants were transferred into fresh tubes and stored frozen at −80 °C. Concentration of proteins was measured using Pierce BCA protein assay kit (Thermo Fisher Scientific) according to manufacturer's instructions. Samples containing 15 µg of protein were mixed with 4 × Laemmli loading buffer with β‐mercaptoethanol and denaturated at 95 °C for 5 min. Next, protein samples were transferred into SDS/PAGE electrophoresis gel. After electrophoresis, proteins were transferred onto the PVDF membrane using Pierce Power Blot Cassette (Thermo Fisher Scientific). After blocking membranes with 5% BSA in TBST (50 mm Tris, 100 mm NaCl, 0.1% Tween 20, pH 7.4) for 1 h at RT, they were incubated overnight at 4 °C with appropriate primary antibodies (1 : 1000 dilution). Following three washes with TBST for 10 min, a secondary anti‐rabbit IgG HRP‐linked goat antibody (1 : 5000 dilution) was added to membranes and the incubation was performed for 1 h at RT. Next, membranes were washed three times for 10 min each with TBST and chemiluminescence signals were visualized with Immobilon Forte Western HRP substrate (Merck Millipore, Burlington, MA, USA) on ChemiDoc Touch Gel Imaging System (Bio‐Rad, Hercules, CA, USA).

### Extracellular oxygen consumption

In brief, rat brown preadipocytes were treated with differentiation medium with or without NPB (100 nm) for 24 h. Thereafter, cells were exposed to adrenaline (1 µm) for 30 min. Extracellular oxygen consumption was measured using Extracellular Oxygen Consumption Assay (Abcam) as we previously descripted [14].

### Statistical analysis

Statistical analysis was conducted using one‐way ANOVA followed by the Bonferroni *post hoc* test. To analyze differences between two groups, *t*‐test was used. Results are presented as mean ± SEM. Statistical significance was considered when *P* < 0.05 (*) or *P* < 0.01 (**). Experiments were performed in duplicates, independently.

## Results

### Expression of *Npbwr1* and neuropeptide B during differentiation of rat primary brown adipocytes

**Fig. 1:**
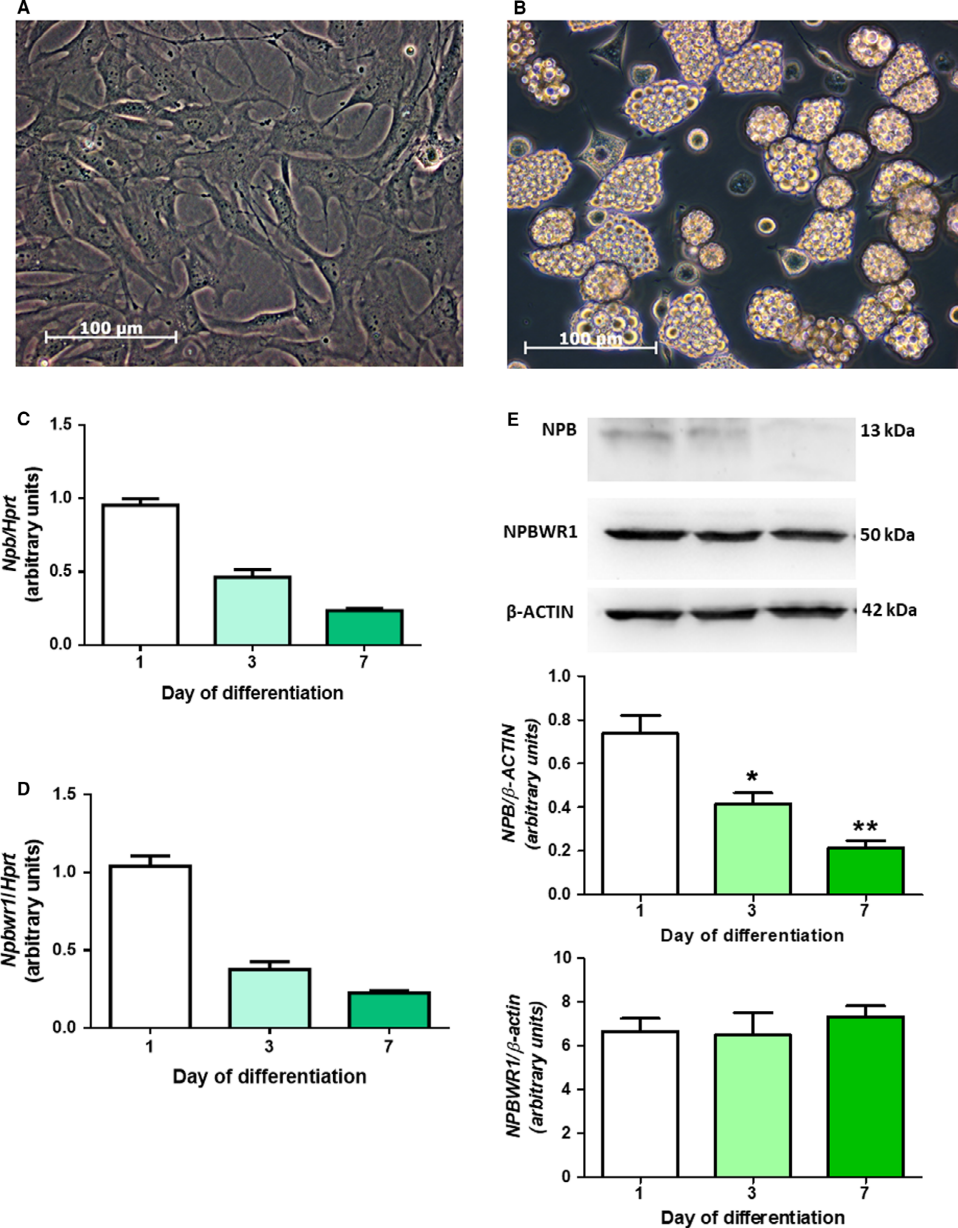
Images of undifferentiated rat primary preadipocytes of brown fat (A) and brown preadipocytes differentiated for 7 days (B; 400×, phase‐contrast, scale bar 100 µm). Expression of NPB (C) and its receptor *Npbwr1* (D) mRNA during differentiation assessed at 1, 3, or 7 days after the onset of differentiation. Western blot detection of NPB and NPBWR1 (E) in cells differentiated for 1, 3, or 7 days. Results are shown as the mean ± SEM [*n* = 6 or 3 (western blots)]. Statistical analysis was conducted using one‐way ANOVA followed by the Bonferroni *post hoc* test. *P < 0.05 or **P < 0.01 vs untreated control.

Representative images of undifferentiated rat brown preadipocytes and brown preadipocytes differentiated for 7 days are presented on Fig. 1A,B. As shown in Fig. 1, rat primary brown preadipocytes express *Npbwr1* (Fig. 1D) and NPB (Fig. 1C) mRNA. The highest level of *Npbwr1* mRNA expression was detected in cells differentiated for 1 day. *Npbwr1* mRNA expression was ~ 2.76‐ and 4.62‐fold lower in cells differentiated for 3 or 7 days, respectively (Fig. 1D). Similarly, the highest mRNA expression of NPB was detected in cells differentiated for 1 day, whereas approximately twofold or fourfold lower expression of NPB mRNA was found in cells differentiated for 3 or 7 days, respectively (Fig. 1C). In addition, we evaluated expression of *NPBWR1* and *NPB* on protein level. As shown in Fig. 1E, *NPBWR1* (50 kDa) protein was present in cells differentiated for 1, 3, or 7 days. *NPBWR1* protein production was stable during differentiation of preadipocytes. Furthermore, we detected *NPB* protein production (13 kDa) in brown fat cells (Fig. 1E). The highest level of *NPB* protein production was detected in cells differentiated for 1 day. *NPB* protein production was ~ 1.77 times and 3.44 times lower in cells differentiated for 3 or 7 days, respectively.

### Neuropeptide B stimulates proliferation and prolongs viability of rat brown preadipocytes

**Fig. 2:**
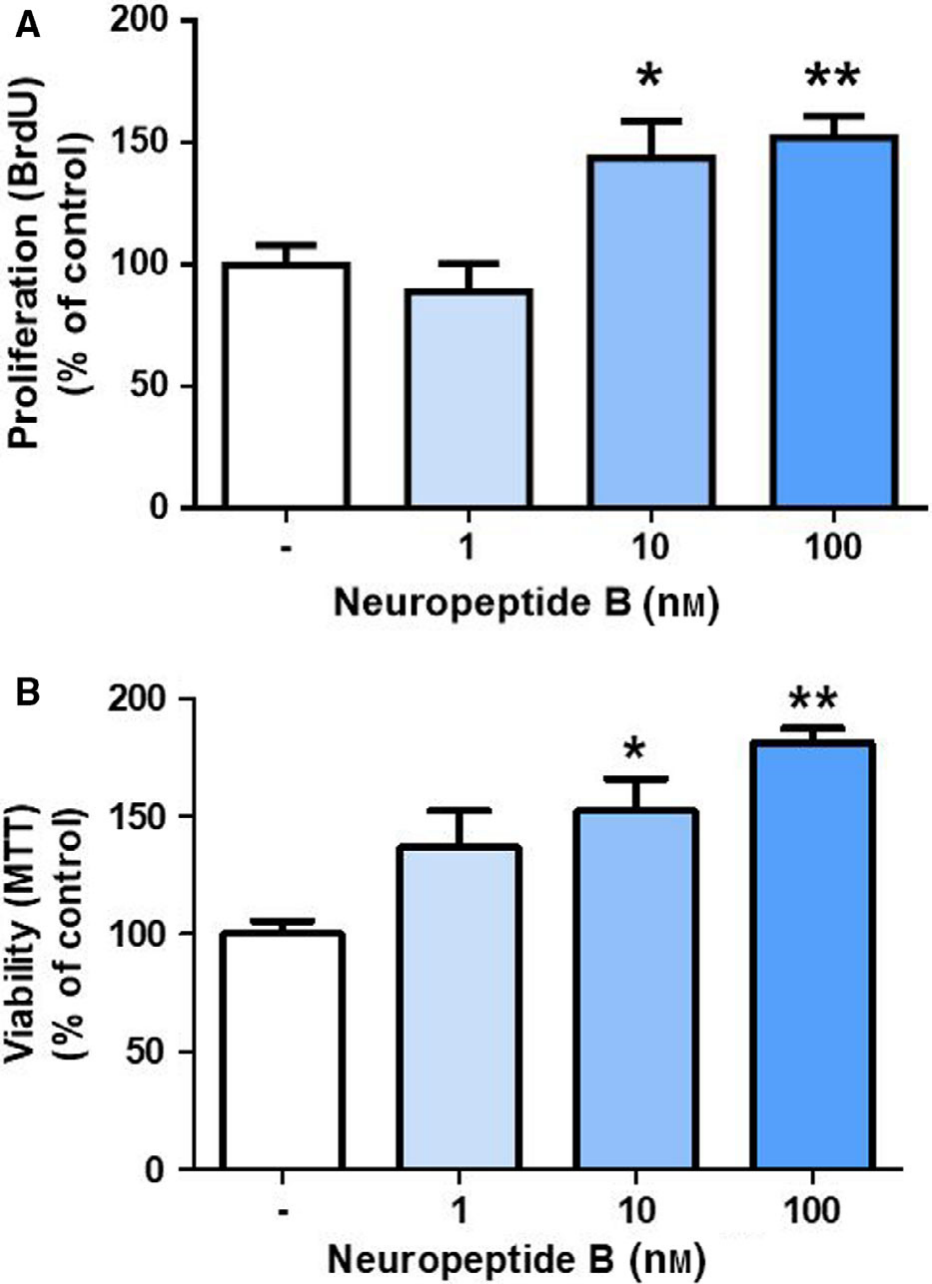
Effect of NPB at 0, 1, 10, or 100 nm on rat brown preadipocytes proliferation and viability. Cell proliferation (A) and viability (B) studied in cells treated with NPB for 24 h. Results are shown as the mean ± SEM (*n* = 8). Statistical analysis was conducted using one‐way ANOVA followed by the Bonferroni *post hoc* test. *P < 0.05 or **P < 0.01 vs untreated control.

Next, we evaluated the effects of NPB on vitality and proliferation of rat brown preadipocytes. As demonstrated in Fig 2A, NPB at 10 and 100 nm increased proliferation of brown preadipocytes after 24 h. By contrast, NPB at 1 nm did not affect cell proliferation. Moreover, neuropeptide (10 or 100 nm) enhanced cell viability assessed in preadipocytes incubated for 24 h (Fig. 2B). These results show that NPB promotes viability and proliferation of rat brown primary preadipocytes.

### Neuropeptide B stimulates differentiation of rat brown preadipocytes into adipocytes

**Fig. 3:**
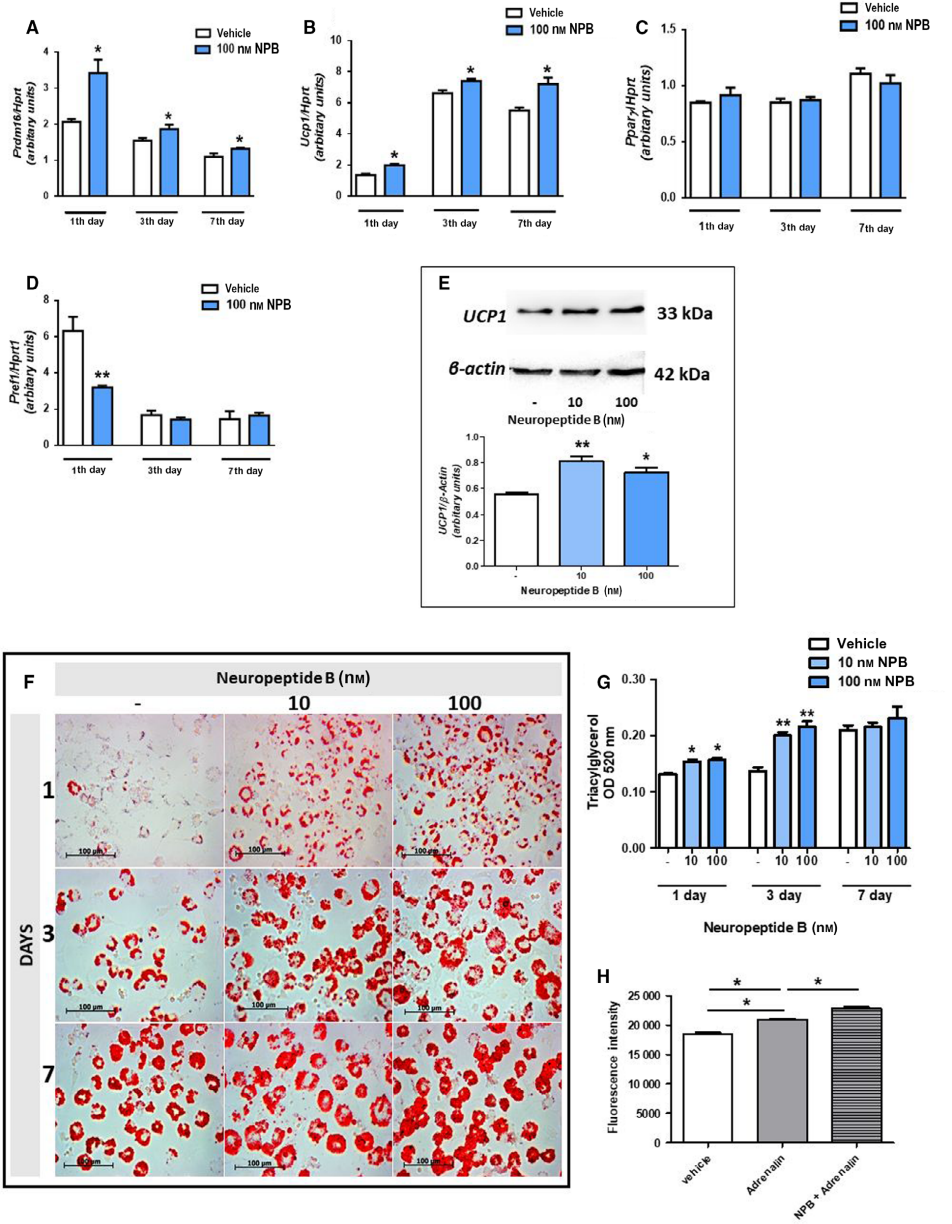
Expression of adipogenic genes mRNA *Prdm16* (A)*, Ucp1* (B), *Pparγ* (C), and *Pref1* (D) in brown preadipocytes differentiated for 1, 3, or 7 days with or without 100 nm NPB. *UCP1* protein production assessed in cells differentiated with or without NPB (10 or 100 nm) (E). Images of brown preadipocytes at 1, 3, and 7 day of culture with (10, 100 nm) or without NPB stained with ORO (F; 400×, scale bar 100 µm) and evaluation of intracellular lipid content (G). Adrenaline‐induced extracellular oxygen consumption evaluated in cells differentiated in the presence or absence of 100 nm NPB (H). Results shown as the mean ± SEM, [*n* = 6 (mRNA expression and ORO), *n* = 3 (western blot)]. Statistical differences were determined by *t*‐test (A‐D) or one‐way ANOVA followed by the Bonferroni *post hoc* test (E, G‐H). *P < 0.05 or **P < 0.01 vs vehicle (water) treated cells.

Since NPB at the concentration of 100 nm was the most efficient in stimulating cell growth, we chose this concentration for subsequent experiments evaluating the effects of NPB on expression of adipogenic markers during differentiation of brown preadipocytes. As shown in Fig. 3A,B, NPB stimulated expression of *Prdm16* and *Ucp1,* and suppressed expression of *Pref1* (Fig. 3D) in preadipocytes differentiated for 1 day. By contrast, NPB failed to affect *Pparγ* mRNA expression assessed 1 day after the onset of differentiation process (Fig. 3C). Furthermore, stimulation of *Prdm16* and *Ucp1* mRNA by NPB was also detected in cells differentiated for 3 days (Fig. 3A,B). However, NPB did not cause any changes in *Pparγ* or *Pref1* mRNA in cells differentiated for 3 days (Fig. 3C,D). As shown in Fig. 3A,B, NPB stimulated mRNA expression of *Prdm16* and *Ucp1* in cells differentiated 7 days; however, it failed to modulate expression of *Pparγ* and *Pref1* mRNA (Fig. 3C,D).

In addition, we studied the effect of NPB on *UCP1* protein production. As shown in Fig. 3E, NPB increased *UCP1* protein production assessed 7 days after the induction of differentiation.

To further characterize the role of NPB in brown adipogenesis, we assessed its effects on lipid accumulation during differentiation of rat preadipocytes. As demonstrated in Fig. 3F,G, NPB at 10 and 100 nm increased intracellular lipid content in preadipocytes differentiated for 1 and 3 days. By contrast, intracellular lipid content was not affected by NPB in cells exposed to differentiation medium for 7 days. Finally, we evaluated extracellular oxygen consumption in cells differentiated with or without NPB (100 nm) and exposed to adrenaline. As demonstrated in Fig. 3H, adrenaline (1 µm) increased extracellular oxygen consumption. In cells differentiated in the presence of NPB, adrenaline‐stimulated oxygen consumption was higher.

In summary, these results show that NPB promotes differentiation of rat brown primary preadipocytes into mature fat cells.

### Neuropeptide B enhances phosphorylation of p38 kinase in rat brown preadipocytes

**Fig. 4:**
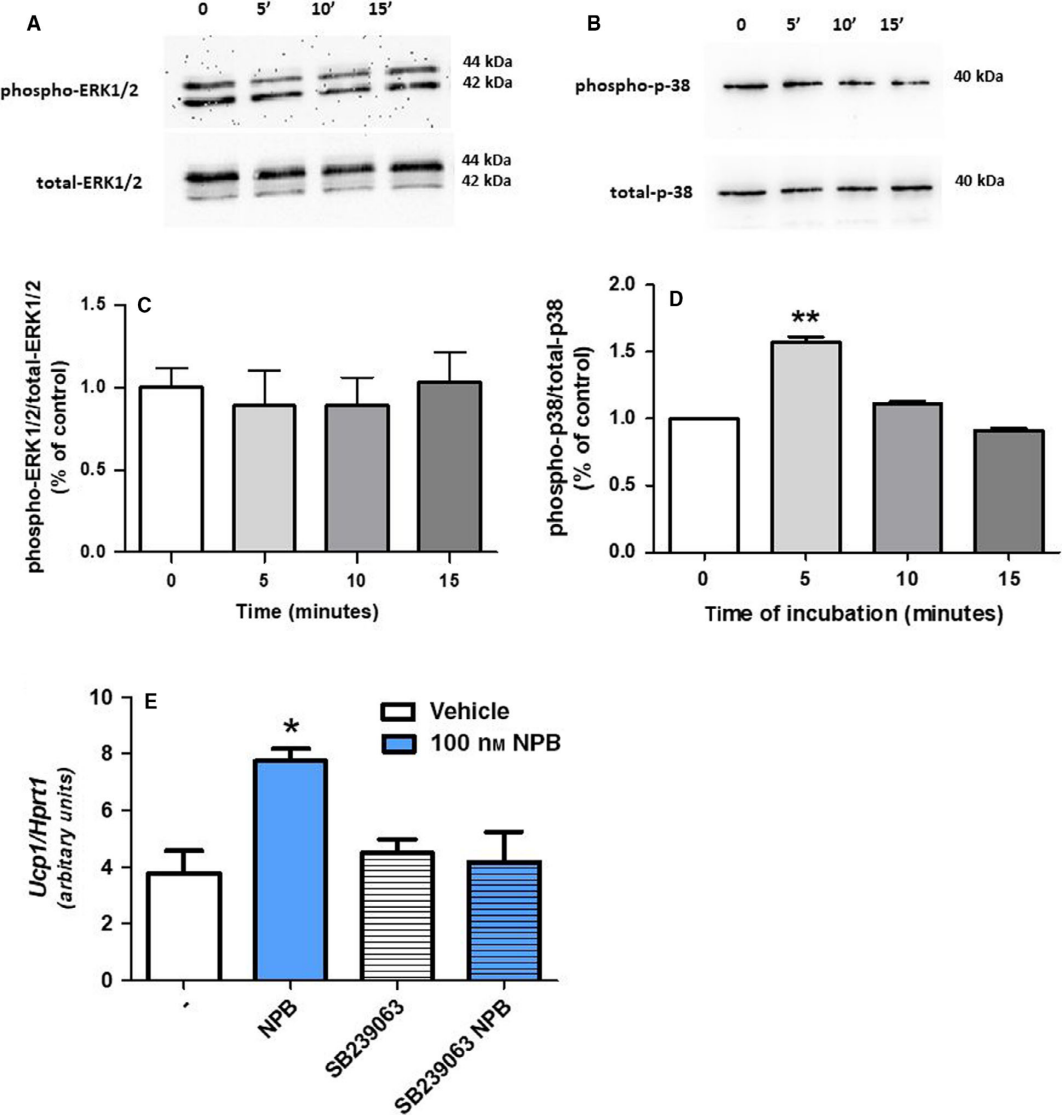
Western blot detection and quantification of ERK1/2 (A‐B) and p38 mitogen‐activated kinase (C‐D) phosphorylation in brown preadipocytes treated with NPB (100 nm) for the indicated time points. Effect of SB239063 blocker (E) on *Ucp1* mRNA expression in cells differentiated with or without NPB (100 nm) for 1 day. Results are shown as the mean ± SEM [*n* = 3 (western blot), *n* = 6 (mRNA expression)]. Statistical differences were determined by one‐way ANOVA followed by the Bonferroni *post hoc* test (B‐E). *P < 0.05 or **P < 0.01 vs vehicle (water) treated cells.

To study potential mechanism, by which NPB promotes differentiation of rat brown preadipocytes into adipocytes, we tested its effects on phosphorylation of p38 and ERK1/2 kinases. As shown in Fig. 4A,B, NPB (100 nm) failed to change phosphorylation of ERK1/2 after 5, 10, or 15 min. On the other hand, NPB (100 nm) increased phosphorylation of p38 in brown fat cells after 5 min (Fig 4C,D). In addition, pharmacological blockade of p38 kinase by SB239063 [17] attenuated NPB‐stimulated *Ucp1* mRNA expression in preadipocytes differentiated for 1 day (Fig. 4E). These results suggested that NPB promotes differentiation of brown preadipocytes via p38 kinase‐dependent mechanism.

**Fig. 5:**
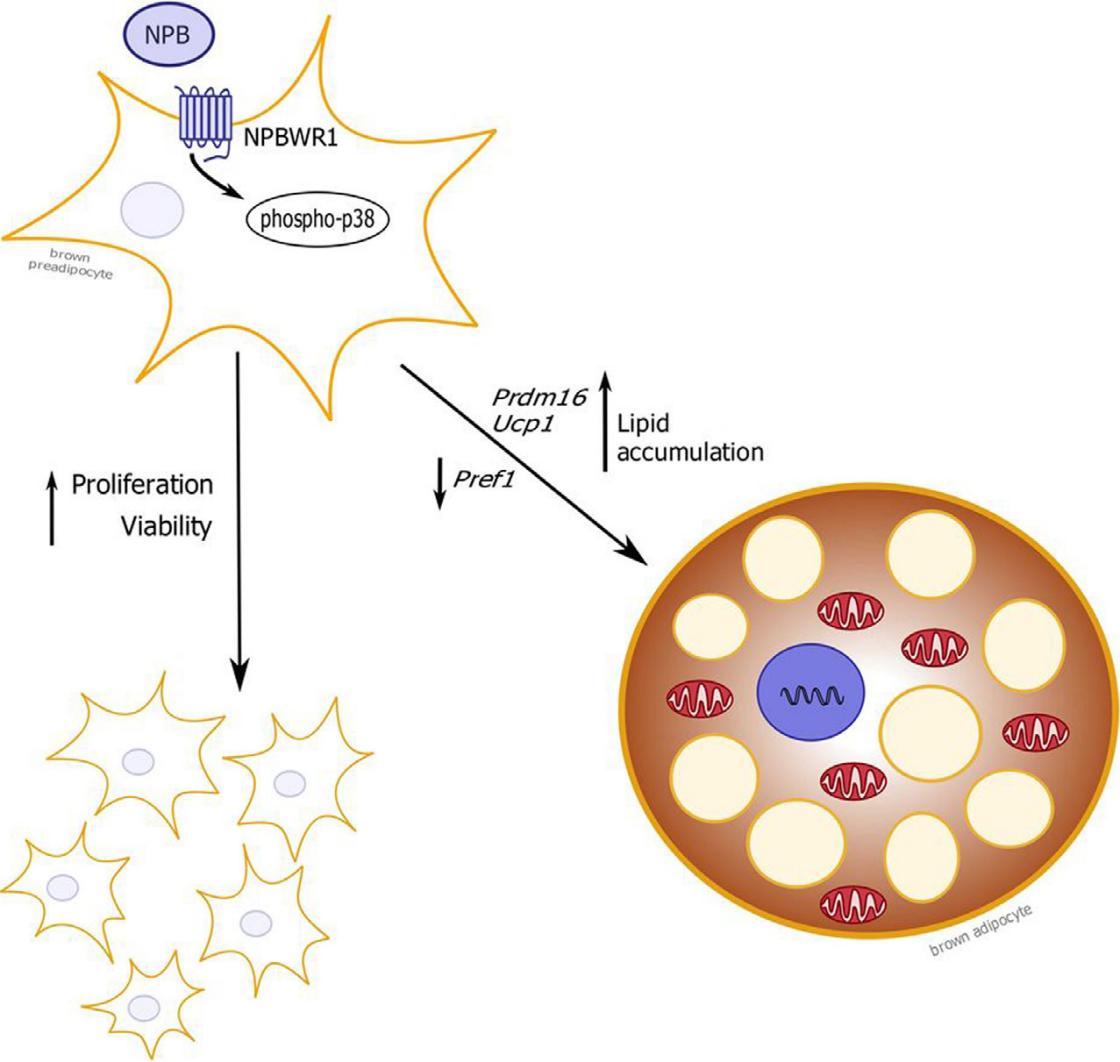
Summary of the effects of NPB in brown preadipocytes.

Summary of the effects of NPB in brown preadipocytes are presented in Fig. 5.

## Discussion

In the present study, we report that NPB promotes proliferation and differentiation of rat brown primary preadipocytes into mature brown fat cells. We found that both NPB and its cognate receptor (NPBWR1) mRNA are expressed in brown preadipocytes. Moreover, we observed that expression of both tested genes is downregulated during differentiation process. Furthermore, we detected *NPBWR1* and *NPB* protein production in brown preadipocytes. However, in contrast to mRNA expression, *NPBWR1* protein production was stable. In this respect, we speculate that translational, but not transcriptional regulation of NPBWR1 may provide an explanation.

These results suggest that NPB may modulate brown preadipocytes functions. The presence of NPB mRNA and protein suggests that this neuropeptide may be involved in modulating brown pre‐ and adipocytes functions via paracrine pathway.

Brown adipogenesis encompasses the processes of enhanced proliferation of brown fat precursor cells and their differentiation into mature brown adipocytes. Therefore, to answer the question addressing a potential role of NPB in controlling brown adipogenesis, we assessed the effects of NPB on cell proliferation as well as viability in brown preadipocytes. In this context, it is important to point out that contradictory observations were reported regarding the effects of NPB on cell replication and cell death. For example, NPB suppresses proliferation of rat calvaria osteoblast‐like cells [18] and promoted replication of rat adrenocortical cells [7]; however, no effects on proliferation in rat insulin‐producing INS‐1E cells were reported [19]. In the present study, we found that NPB promotes both viability and proliferation of brown preadipocytes. Therefore, even though the effects of NPB on cell proliferation appear to be cell specific, our data indicate that by acting on brown fat precursor cells, NPB may increase a fraction of preadipocytes, which is able to differentiate into mature brown adipocytes.

Next, we assessed the effects of NPB on differentiation of rat brown preadipocytes into brown adipocytes. To address this question, we studied the effects of NPB on expression of main proadipogenic factors as well as markers of brown preadipocytes differentiation such as *Prdm16*, *Ucp1,* and *Pparγ* [20]. It is important to note that both PRDM16 and PPARγ increased UCP1 expression [21, 22]. Moreover, we evaluated the expression of *Pref1,* which is known to suppress brown adipogenesis [23]. Our results demonstrate that NPB promotes expression of *Prdm16* and *Ucp1* mRNA, without affecting *Pparγ* mRNA expression. Furthermore, we detected that NPB promotes *UCP1* protein production.

Since we found that NPB promotes expression *Ucp1* mRNA expression, the inability of NPB to increase *Pparγ* mRNA is rather unexpected. However, it needs to be pointed out that NPB is able to stimulate *Prdm16* mRNA expression in our current study. Interestingly, it was found that overexpression of PRDM16 is able to promote *Ucp1* mRNA expression even in the absence of PPARγ [21]. Furthermore, depletion of PPARγ in adipose tissue in mice had no effect on UCP1 expression in brown adipose tissue [24]. Overall, these results suggest that NPB promotes differentiation of rat brown preadipocytes into mature fat cells independently on PPARγ expression.

To confirm proadipogenic effects of NPB in brown preadipocytes, we studied its effects of intracellular lipid accumulation. Of note, intracellular lipid content increases during differentiation of brown preadipocytes [25]. Our data show that NPB increases intracellular lipids in preadipocytes during the differentiation. However, it needs to be noted that these effects were observed only in cells exposed to NPB for 1 or 3 days but not after 7 days. In line with these findings, we found that the most prominent effect of NPB on stimulation of *Prdm16* (proadipogenic) and downregulation of *Pref1* was also detected in cells exposed to NPB for 1 day. In this context, it needs to be considered that a prolonged exposition of GPCRs to their ligands may cause receptor desensitization [26]. Thus, it is possible that prolong incubation of brown preadipocytes in the presence on NPB leads to attenuation of biological effects of this peptide on adipogenesis *in vitro*.

Brown adipogenesis is a complex process, which is influenced by a variety of environmental and biological factors [27, 28]. There is evidence showing that stimuli of MAP kinases such as p38 and ERK1/2 kinases can promote thermogenesis as well as formation of brown fat cells [29, 30]. For example, it was demonstrated that p38 promotes the expression and/or activity of transcription factors such as PGC‐1α, activating transcription factor 2; both of which are of crucial relevance at stimulating UCP1 expression [29]. Importantly, NPB was found to enhance ERK1/2 phosphorylation in INS‐1E cells [19]. Therefore, since NPB is able to increase differentiation of rat preadipocytes into mature adipocytes in our study, we assessed the effects of this peptide on phosphorylation of p38 and ERK1/2. Here, we found that NPB stimulates phosphorylation of p38 kinase, while it has no effect on ERK1/2 phosphorylation. Keeping in mind an importance of p38 kinase at the modulation of brown adipogenesis, we assessed the consequences of its pharmacological blockade by SB239063 [17] on NPB ‐stimulated *Ucp1* mRNA expression. Suppression of p38 activity by SB239063 abolished the stimulatory effect of NPB on *Ucp1* mRNA expression. Mechanism underlying p38 activation by NPB remains unknown. P38 activation can be induced by a variety of upstream signals such as the MAP kinase kinases [31]. However, there is also evidence indicating that p38 activity can be modulated by PKC [32, 33]. In this context, it is worth to point out that also neuropeptide W, another NPBWR1 ligand, can stimulate the proliferation as well as early differentiation of murine chondrocytes via a PKC‐dependent mechanism [34]. Thus, it cannot be excluded that NPB ‐stimulated p38 depends upon PKC activation.

In summary, these results provide evidence that NPB promotes differentiation of brown adipocytes via p38 but not ERK1/2‐dependent mechanism.

Our study has several limitations. First of all, we studied the effects of NPB on brown adipogenesis *in vitro*, only. Therefore, the results of our study should be interpreted cautiously. Nevertheless, as we already mentioned, NPB or NPBWR1‐deficient mice have features consistent with adult‐onset obesity as well as impaired energy expenditure [8, 10], which is a hallmark of brown fat tissue deficiency [35, 36]. However, it remains unknown whether these metabolic abnormalities result from an impaired brown adipogenesis. Thus, the effects of NPB on brown fat tissue formation and activity need to be studied *in vivo*. Furthermore, it needs to be elucidated whether NPB is able to modulate proliferation and/or differentiation of human brown fat precursor cells.

In conclusion, our study demonstrated that neuropeptide independently can promote proliferation and differentiation of rat brown preadipocytes into mature brown fat cells.

## Conflict of interest

The authors declare no conflict of interest.

## Author contributions

TW and MS designed the study and wrote the manuscript; TW, MB, PD, MS, and DS involved in collection, analysis, and interpretation of data; TW and MS performed the decision to submit the article for publication; MZS, KWN, and OW edited and revised the manuscript.

## Data Availability

The data that support the findings of this study are available from the corresponding author (marek.skrzypski@up.poznan.pl) upon reasonable request.
